# Inferring species trees from incongruent multi-copy gene trees using the Robinson-Foulds distance

**DOI:** 10.1186/1748-7188-8-28

**Published:** 2013-11-01

**Authors:** Ruchi Chaudhary, John Gordon Burleigh, David Fernández-Baca

**Affiliations:** 1 Department of Computer Science, Iowa State University, Ames, IA 50011, USA; 2 Department of Biology, University of Florida, Gainesville, FL 32611, USA

## Abstract

**Background:**

Constructing species trees from multi-copy gene trees remains a challenging problem in phylogenetics. One difficulty is that the underlying genes can be incongruent due to evolutionary processes such as gene duplication and loss, deep coalescence, or lateral gene transfer. Gene tree estimation errors may further exacerbate the difficulties of species tree estimation.

**Results:**

We present a new approach for inferring species trees from incongruent multi-copy gene trees that is based on a generalization of the Robinson-Foulds (RF) distance measure to multi-labeled trees (mul-trees). We prove that it is NP-hard to compute the RF distance between two mul-trees; however, it is easy to calculate this distance between a mul-tree and a singly-labeled species tree. Motivated by this, we formulate the RF problem for mul-trees (MulRF) as follows: Given a collection of multi-copy gene trees, find a singly-labeled species tree that minimizes the total RF distance from the input mul-trees. We develop and implement a fast SPR-based heuristic algorithm for the NP-hard MulRF problem.

We compare the performance of the MulRF method (available at http://genome.cs.iastate.edu/CBL/MulRF/) with several gene tree parsimony approaches using gene tree simulations that incorporate gene tree error, gene duplications and losses, and/or lateral transfer. The MulRF method produces more accurate species trees than gene tree parsimony approaches. We also demonstrate that the MulRF method infers in minutes a credible plant species tree from a collection of nearly 2,000 gene trees.

**Conclusions:**

Our new phylogenetic inference method, based on a generalized RF distance, makes it possible to quickly estimate species trees from large genomic data sets. Since the MulRF method, unlike gene tree parsimony, is based on a generic tree distance measure, it is appealing for analyses of genomic data sets, in which many processes such as deep coalescence, recombination, gene duplication and losses as well as phylogenetic error may contribute to gene tree discord. In experiments, the MulRF method estimated species trees accurately and quickly, demonstrating MulRF as an efficient alternative approach for phylogenetic inference from large-scale genomic data sets.

## Background

With the proliferation of next generation sequencing technologies, there is great interest in using large genomic data sets for phylogenetic inference. One challenge for such phylogenomic analyses is that the genes sampled from the same set of species often produce conflicting trees [1]. Some of the incongruence among trees may be due to errors in the phylogenetic analyses. Alternately, the discordance may reflect biological processes such as recombination, gene duplication, gene loss, deep coalescence, or lateral gene transfer (LGT) [1-6]. Thus, in order to construct phylogenetic hypotheses from genomic data, it is necessary to address the incongruence among gene trees. Furthermore, any method for such phylogenetic analyses also must be computationally tractable for extremely large genomic data sets.

Constructing species phylogenies from a collection of gene trees requires summarizing and reconciling the phylogenetic information contained in the genes. The majority of such species tree reconstruction methods reconcile the gene tree and species tree topologies using an optimality criterion based on a specific evolutionary process, such as gene duplication and loss or deep coalescence. In this paper we consider the problem of constructing species tree from gene trees using a tree distance measure that is not based on a specific biological process. We evaluate how our method performs in gene tree simulation experiments and with a large genomic data set from plants.

Existing methods for inferring species trees from collections of gene trees can be divided into two broad categories: non-parametric methods based on gene tree parsimony (GTP), and parametric methods that use likelihood (e.g., [7,8]) or Bayesian frameworks (e.g., [9-11]). GTP methods take a collection of discordant gene trees and try to find the species tree that implies the fewest evolutionary events. GeneTree [12], DupTree [13], and DupLoss [14] seek to minimize the number of duplications or duplications and losses. GeneTree [12], Mesquite [1], PhyloNet [15], and the method of [14] minimize deep coalescence events. The Subtree Prune and Regraft (SPR) supertree method [16] is based on minimizing the number of LGT events, and thus, it also can be considered a GTP method. Some of these methods have fast and effective heuristics, enabling the analysis of very large data sets. However, errors in the gene trees can mislead GTP analyses [17-19]. Furthermore, in some cases GTP methods may be statistically inconsistent, even when the gene tree topologies are correct [20]. Parametric methods exist based on either coalescence [7,10] or gene duplication and loss models [8]. Although such approaches have a strong statistical foundation, they can be extremely computationally expensive.

While the existing methods differ widely in their details, with the exception of [9], they are based on assumptions about the specific biological cause of discordance among gene trees. For example, GTP methods based on a duplication and loss cost implicitly assume that the differences between a gene tree and the species tree are caused by either gene duplications or losses. This does not necessarily mean that these methods will fail when their assumptions are incorrect, but it suggests that it is important to explore a range of different objectives for reconciling gene trees.

We present a new approach for constructing a species tree from discordant multi-copy gene trees based on a generic, non-biological distance measure. Our distance measure generalizes the Robinson-Foulds (RF) distance measure to multi-labeled trees (mul-trees) or trees where multiple leaves can have the same label. Our method takes as input a collection of multi-copy gene trees (mul-trees) and finds a species tree at minimum RF distance to the input gene trees. Our contributions are as follows: 

•We study the problem of computing the RF distance between two mul-trees, and show that it is NP-complete (Theorem 1).

•We formulate an RF problem for mul-trees (MulRF) that takes a collection of multi-copy gene trees as input and constructs a binary species tree that is at minimum RF distance from each input gene tree (Section The MulRF Problem). A key component of this approach is a simple and efficient technique to compute the RF distance between an input multi-copy gene tree and a *singly-labeled* species tree. (Note the contrast with the previously-mentioned NP-completeness result.)

•MulRF is an NP-hard problem, so heuristics are required to estimate solutions for large data sets. We provide a fast *Θ*(*nmk*)-time algorithm for the MulRF problem, where *n* is the total number of distinct leaf labels in the input collection of gene trees, *m* is the sum of *n* and the number of gene sequences in a input gene tree (assuming for convenience that all the input gene trees are built on approximately the same number of gene sequences), and *k* is the number of input gene trees (Section Solving the MulRF problem).

•We implemented the MulRF heuristic and examined its performance on simulated gene tree data sets that incorporate gene tree error, gene duplication and loss, and/or lateral gene transfer and a data set of nearly 2000 plant gene trees (Section Experimental evaluation).

We note that there has been much recent work on mul-trees ranging from constructing strict and majority rule consensus mul-trees to deriving diameter bounds for various metrics on mul-trees (see, [21-26]). Further, various problems related to RF distance have received attention. The RF distance has been extended to increase its robustness without sacrificing polynomial time computability [27,28]. These methods appear to work well when both input trees are singly-labeled, but there are no direct extensions of them for mul-trees. Alternatively, RF distance has been used in the supertree method for singly-labeled input trees [29,30] and the maximum-likelihood supertree approach of [31]. Here, we use RF distance for building species trees from mul-trees, which allows us to incorporate a wealth of genomic data from multi-copy genes into phylogenetic inference.

Our heuristic algorithm for MulRF problem shares several core concepts with unrooted RF supertree (URF) algorithm of [30], but there are theoretical and practical differences. In particular, our local search heuristic of MulRF is based on the SPR operation, unlike the *p*-Edge Contract and Refine operation (*p*-ECR) used for URF [30]. Typically, the SPR operation is more effective in exploring the space of trees compared to *p*-ECR operation; this also enables the MulRF heuristic to run as a standalone application on the given gene trees, independent from the rooted RF heuristic of [29]. In contrast, the *p*-ECR-based URF algorithm of [30] uses the output of the rooted method of [29] as a starting tree.

We performed gene tree simulation experiments to evaluate the accuracy of our method by comparing it against the model species tree used to simulate the data. We compared the species trees constructed by MulRF and GTP methods that consider only duplication [13], duplication and loss [14], and only LGT [16] with the true species trees. Our simulated data sets were too large to analyze with parametric methods, so we were unable to compare MulRF with these approaches. For example, when we ran Phyldog [8] on a single 50-taxon, 400 gene data set using 4 cores, it did not converge on a species tree in 110 hours. In contrast, MulRF gave an answer within a few seconds.

In all experiments, MulRF produced trees that are more similar to the true species trees than those obtained by the three GTP methods. This suggests that MulRF may be more robust than GTP methods to complex processes of gene evolution, including LGT, and in the presence of gene tree error. Furthermore, our algorithm runs quickly on moderate-size data sets, finishing in under two minutes on data sets containing 300 gene trees evolved over 100 taxon species trees. This suggests it is scalable for large-scale phylogenomic analyses. Finally, we examined the performance of the MulRF method on an unpublished plant gene tree data set with nearly 2000 gene trees from 22 species. The resulting species tree from the MulRF method was largely consistent with published plant phylogenies.

## Preliminaries

Let *X* be a finite set of *labels*. A *phylogenetic mul-tree on X* (or *mul-tree*, for short) is a pair T=(T,φ) consisting of an unrooted tree *T*, whose leaf set is denoted by ℒ(T), and where every internal vertex has degree at least three, along with a surjective map φ:ℒ(T)→X. The tree *T* is called the *underlying tree* of  and *φ* is called the *labeling map* of . We say that  is a *singly-labeled tree* if *φ* is a bijection between ℒ(T) and *X* (i.e., |*φ*^-1^(*x*)|=1 for all *x*∈*X*). Singly-labeled trees are also referred to as *phylogenetic X-trees* ([32]; page 17).

A mul-tree T=(T,φ) is *binary* if every internal vertex of *T* has degree 3. A vertex of *T* is said to be *unresolved* if its degree is greater than three. We use *V*(*T*) and *E*(*T*) to denote the set of vertices and the set of edges of *T*. The set of all internal vertices of *T* is I(T):=V(T)∖ℒ(T). The *size* of , denoted by |T|, is the number of elements in ℒ(T).

Let T=(T,φ) be a mul-tree on *X* and *U*⊆*X*. Let *T*[ *U*] denotes the minimum subtree of *T* induced by the elements of {v∈ℒ(T):φ(v)∈U}. The *restriction of T to *, denoted *T*_|*U*_ is the tree obtained from *T*[ *U*] by suppressing all vertices of degree two. The *restriction of φ to U*, denoted *φ*_|*U*_ is the surjective mapping $\varphi_{|U}:ℒ(T_{|U})\to U$, where for each $v\in ℒ(T_{|U}),\varphi_{|U}(v)=\varphi (v)$. The *restriction* of  to *U*, denoted by $T_{|U}$, is the mul-tree on *U* given by $T_{|U}=(T_{|U},\varphi_{|U})$.

Two mul-trees $T_{1}=(T_{1}=(V_{1},E_{1}),\varphi_{1})$ and $T_{2}=(T_{2}=(V_{2},E_{2}),\varphi_{2})$ on *X* are isomorphic if there exists a bijection *τ*:*V*_1_→*V*_2_, which induces a bijection between *E*_1_ and *E*_2_, subject to the condition that *φ*_1_(*u*)=*φ*_2_(*τ*(*u*)) for all $u\in ℒ(T_{1})$.

**Figure 1:**
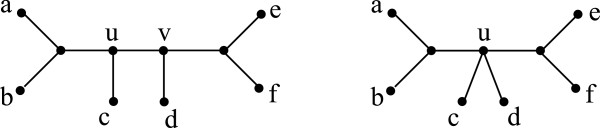
**Contraction and refinement.** Contracting edge {*u,v*} in the mul-tree on the left produces the mul-tree on the right. Conversely, refinement of vertex *u* in the mul-tree on the right produces the mul-tree on the left.

We define two basic operations on a mul-tree (*T*,*φ*). The *contraction* of an internal edge of *T* collapses that edge and identifies its two endpoints, yielding a new tree *T*^′^ and a corresponding mul-tree (*T*^′^,*φ*). (Note that, since *T*^′^ and *T* have the same leaf sets, *φ* is also defined on *T*^′^.) Let *v* be an unresolved vertex of *T*. A *refinement* of *v* is obtained by partitioning the set of neighbors of *v* into two sets *N*_1_ and *N*_2_, such that |*N*_1_|,|*N*_2_|>1, replacing *v* by two vertices *v*_1_ and *v*_2_ connected by an edge, and making the vertices of *N*_1_ neighbors of *v*_1_ and those in *N*_2_ neighbors of *v*_2_. This yields a new tree *T*^′^, with the same leaf set as *T*, and a corresponding mul-tree (*T*^′^,*φ*). Contraction and refinement can be viewed as inverses of each other (Figure 1).

Let $T_{1}=(T_{1},\varphi_{1})$ and $T_{2}=(T_{2},\varphi_{2})$ be mul-trees on *X*_1_ and *X*_2_, respectively, such that *X*_1_∩*X*_2_≠*∅*. We say that $T_{1}=(T_{1},\varphi_{1})$ and $T_{2}=(T_{2},\varphi_{2})$ have *matching label multiplicities* if $\left|\varphi_{1}^{-1}(x)|=|\varphi_{2}^{-1}(x)\right|$ for all *x*∈*X*_1_∩*X*_2_. The *Robinson-Foulds (RF)* distance between two mul-trees $T_{1}$ and $T_{2}$ with identical label sets and matching label multiplicities, denoted by $\text{RF}(T_{1},T_{2})$, is defined as the minimum number of contractions and refinements necessary to transform $T_{1}$ into another mul-tree isomorphic to $T_{2}$[33,34]. (Note that [33] originally defined their distance measure for singly-labeled trees. Later on, [34] showed that the definition extends naturally to mul-trees.) We extend the RF distance to mul-trees $T_{1}$, on *X*_1_, and $T_{2}$, on *X*_2_, with *X*_1_⊆*X*_2_ and matching label multiplicities, as $\text{RF}(T_{1},T_{2}):=\text{RF}(T_{1},{T_{2}}_{|X_{1}})$.

Let T=(T,φ) be a mul-tree on *X*. Let *M* be a multiset on *X* such that the multiplicity in *M* of each element *x*∈*X* is |*φ*^-1^(*x*)|. A *splitA*|*B* of  is a bipartition of *M*, i.e., the sum of multiplicities of each element *x*∈*X* in *A* and *B* is equal to the multiplicity of *x* in *M*. Multisets *A* and *B* are the *parts* of split *A*|*B*. (Note that if  is singly-labeled, then *M*, *A*, and *B* are sets.) The set of all splits induced by the internal edges of a mul-tree  is denoted by Σ(T).

**Figure 2:**
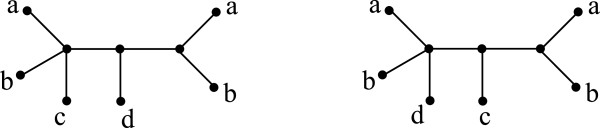
**Contradicting example.** Two mul-trees that induce the same set of splits but are not isomorphic. From ([23], Figure one).

As Figure 2 illustrates, two mul-trees $T_{1}$ and $T_{2}$ such that $\Sigma (T_{1})=\Sigma (T_{2})$ may not be isomorphic. (See also ([34], Figure five) for a larger example.) On the other hand, by the Splits Equivalence Theorem ([32]; p. 44), if $T_{1}$ and $T_{2}$ are singly-labeled trees, then $\Sigma (T_{1})=\Sigma (T_{2})$ implies that $T_{1}$ and $T_{2}$ are isomorphic. Further, in this case, [33].

(1) $$\text{RF}(T_{1},T_{2})=|(\Sigma (T_{1})\setminus \Sigma (T_{2}))\cup (\Sigma (T_{2})\setminus \Sigma (T_{1}))|$$

Since mul-trees do not satisfy the Splits Equivalence Theorem, the RF distance between two of them cannot be computed by splits using expression (1). Nevertheless, as we show in Section The MulRF Problem, the formula will be useful for computing the RF distance between input gene tree and a species tree.

Ganapathy et al. [34] gave a worst-case exponential time algorithm for computing the RF distance between two mul-trees. The next result suggests that a polynomial time algorithm is unlikely.

### Theorem 1

Computing the RF distance between two mul-trees is NP-complete.

### Proof

See the Additional file 1. □

## The MulRF Problem

A *profile*$P=(T_{1},T_{2},\ldots ,T_{k})$ is a tuple of mul-trees, also called *input mul-trees*, representing multi-copy gene trees, where, for each $i\in \{1,\ldots ,k\},T_{i}$ has label set *X*_*i*_. A *species tree* for  is a singly-labeled phylogenetic tree  on *Y*, where $Y=\overset{k}{\underset{i=1}{⋃}}X_{i}$.

A species tree  for  and a tree  in  will not, in general, have matching label multiplicities, since  is singly-labeled, while  need not be. In order to define RF(T,S), we will extend the species tree to add the missing duplicate leaf labels, thereby converting it into a mul-tree. We explain this formally next.

**Figure 3:**
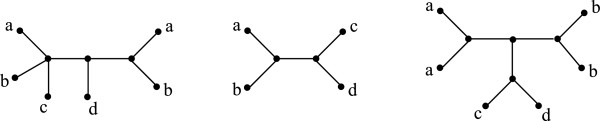
**Rooting an unrooted tree.** Phylogenetic tree **T** with leaf label set {*a, b, c, d, e*}. The rooted phylogenetic tree  with *r*=*a* is also shown.

Let T=(T,φ) be an input mul-tree on *X* and S=(S,ϕ) be a species tree on *Y*; thus, *X*⊆*Y*. The *extension of* relative to *is a mul-tree*S*=(S*,ϕ*) on *Y*, constructed from  by doing the following for each vertex s∈ℒ(S) such that |*φ*^-1^(*ϕ*(*s*))|>1. Let *k*:=|*φ*^-1^(*ϕ*(*s*))|. Replace *s* by an internal vertex connecting to *k* leaves {*l*_1_,…,*l*_*k*_} labeled with *ϕ*(*s*); i.e., ∀*i*(1≤*i*≤*k*),ϕ*(*l*_*i*_) = *ϕ*(*s*). See Figure 3. We now define RF(T,S) to be RF(T,S*), where S* is the extension of  relative to . We define the *RF distance from a profile**to a species tree* for  as $\text{RF}(P,S):=\underset{T\in P}{\sum }\text{RF}(T,S)$.

Let ℬ(P) be the set of all binary species trees for .

### Problem 1

(**RF for MUL-Trees (MulRF)**)Instance: A profile $P=(T_{1},T_{2},\ldots ,T_{k})$ of mul-trees.Find: A species tree $S^{⋆}$ for  such that $\text{RF}(P,S^{⋆})=\underset{S\in ℬ(P)}{\min}\text{RF}(P,S)$.

Observe that the solution to the MulRF problem may not be unique. Further, the MulRF problem is NP-hard even when all the input mul-trees are singly labeled and their leaf label sets are identical [35]. Nevertheless, the “small” version of the problem —computing the RF distance between a profile of mul-trees and a species tree— is easy to solve. For each input mul-tree , we (i) construct the extension S* of the species tree relative to ; (ii) differentiate duplicate leaf labels in both S* and ; and (iii) apply the split-based formula (1) to compute the RF distance between the resulting singly-labeled phylogenetic trees. Next, we explain this process formally.

A *full differentiation* of a mul-tree T=(T,φ) on *X* is a singly-labeled tree **T**=(*T*,*φ*^′^) on *X*^′^[34]. Note that both  and **T** have identical underlying trees, but the labeling map is surjective in the former, and bijective in the latter. Thus, *X* and *X*^′^ may be different sets and |*X*|≤|*X*^′^|. Intuitively, a full differentiation of a mul-tree differentiates the leaves that have identical leaf labels.

Let T=(T,φ) and S=(S,ϕ) be two mul-trees, on *X* and *Y*, respectively, such that  and  have matching label multiplicities. Two full differentiations **T**=(*T*,*φ*^′^) and **S**=(*S*,*ϕ*^′^) of  and , respectively, are *consistent* if for each *a*∈*X*∩*Y*,*φ*^′^(*φ*^-1^(*a*))=*ϕ*^′^(*ϕ*^-1^(*a*)), i.e, both **T** and **S** have same set of new leaf labels for each common leaf label in  and . For instance, a consistent full differentiation can be obtained by relabeling each of the *k* copies of each leaf label *a* by *a*_1_,*a*_2_,…,*a*_*k*_ in both the mul-trees.

### Theorem 2 ([34])

Let T=(T,φ) and S=(S,ϕ) be mul-trees with matching label multiplicities. Then, RF(T,S)=min{RF(T,S):T and **S** are mutually consistent full differentiations of  and , respectively }.

We can prove the following result.

### Theorem 3

Let  be a mul-tree in a profile  and let  be a species tree for . Let S* be the extension of  relative to . Then, for each pair of consistent full differentiations (**T**_1_,**S**_1_) and (**T**_2_,**S**_2_) of  and S* we have RF (**T**_1_,**S**_1_)= RF (**T**_2_,**S**_2_).

### Proof

Let T=(T,φ) be the input mul-tree on *X*. We prove the theorem by showing that for each *a*∈*X*, where |*φ*^-1^(*a*)|=*k*, all *k*! ways of uniquely relabeling corresponding *k* leaves in both  and S* result into the same number of matched and unmatched splits in the corresponding mutually consistent full differentiations. The set of splits in  can be divided into two categories: □

•*Category 1:* Splits that have all the leaves labeled with *a* in one part. Such a split will always have a match, irrespective of the labeling.

•*Category 2:* The remaining splits. Such splits are not present in S*, therefore, they will never have a match, irrespective of the labeling.

Thus, we can compute the RF distance between an input phylogenetic mul-tree and a species tree by computing the RF distance between *any* consistent full differentiations of the two trees. Since these full differentiations are singly-labeled trees, the RF distance between them can be found using Equation (1).

## Solving the MulRF problem

**Figure 4:**
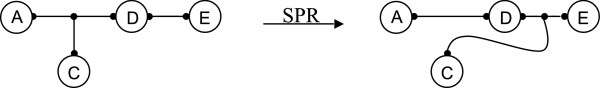
**Species tree extension.** From left to right, input mul-tree , the species tree , and the mul-tree S*. S* is the extension of  relative to .

Our local search heuristic for the MulRF problem starts with an initial (singly-labeled) species tree and explores the space of possible species trees in search of a *locally optimum* species tree, a species tree for  whose score is minimum within its “neighborhood”. The neighborhood is defined in terms of the *Subtree Prune and Regraft* (*SPR*) operation [36]. An SPR operation on an unrooted, binary, singly-labeled phylogenetic tree T=(T,φ) on *X* cuts any edge *e*∈*E*(*T*), thereby pruning a subtree *t*, and then regrafts *t* by the same cut edge to a new vertex obtained by subdividing a pre-existing edge in *T*-*t* (Figure 4). The resulting phylogenetic tree is said to be *obtained from**by a single SPR operation*. The set of all phylogenetic trees obtained by the application of a single SPR operation on  is called the *SPR neighborhood* of , and is denoted by $\text{SP}R_{T}$.

### Problem 2

(**SPR Search**) Instance: A profile $P=(T_{1},T_{2},\ldots ,T_{k})$ of mul-trees and a binary species tree  for . Find: A species tree $S^{⋆}$ for  such that $S^{⋆}\in \text{SP}R_{S}$ and $\text{RF}(P,S^{⋆})=\underset{S^{\prime }\in \text{SP}R_{S}}{\min}\text{RF}(P,S^{\prime })$.

The SPR Search based MulRF algorithm runs in two phases. In phase I, the algorithm quickly builds a likely suboptimal initial species tree using a greedy leaf adding procedure. This procedure first builds a phylogenetic tree on three randomly selected labels, and then it adds the remaining labels one at a time in a randomized order. In phase II, the algorithm performs a series of SPR Search iterations, each of which starts with an initial species tree and the input mul-trees. The output species tree of one SPR Search iteration serves as the initial species tree for the next iteration. When the resulting species tree of an SPR Search iteration is same as its initial species tree (i.e., there is no improvement in the score), the MulRF algorithm stops and returns the initial species tree of that iteration as the output.

Let the size of the input species tree  for the SPR Search problem be *n*, i.e. n:=|S|. For each T∈P, let m:=|T|+|S|. (For convenience, we assume that all the input gene trees have approximately the same size.) Let *k* be the number of input gene trees in . In Section Solving the SPR search problem, we present an algorithm for the SPR search problem that runs in time *Θ*(*nmk*). (More precisely, if the size of the *i*th gene tree in the input profile be *t*_*i*_ then the complexity of our algorithm is $\Theta (\overset{k}{\underset{i=1}{\sum }}n(n+t_{i}))$. We made the assumption about the size of the input gene trees to simplify this complexity.) The algorithm relies on results from [30], which characterize the RF distance between unrooted phylogenetic trees in terms of least common ancestors in rooted versions of those phylogenies. These properties enable us to update the RF distance quickly after an SPR operation has been applied to one of the trees. For completeness, we briefly review these results in the next subsection. For a full discussion with proofs, see [30].

### Robinson-Foulds distance and least common ancestors

In this subsection, we deal exclusively with singly-labeled trees, which we refer to simply as *phylogenetic trees*.

A phylogenetic tree T=(T,φ) is *rooted* if the underlying tree *T* is rooted; this means that *T* has exactly one distinguished vertex *rt*(*T*), called the *root*. A rooted phylogenetic tree is *binary* if the root has degree two and every other internal vertex has degree three.

Let T=(T,φ) be a rooted phylogenetic tree on *X*. A vertex *v* of *T* is *internal* if v∈V(T)∖(ℒ(T)∪rt(T)). The set of all internal vertices of *T* is denoted by *I*(*T*). We define ≼_*T*_ to be the partial order on *V*(*T*) where *x*≼_*T*_*y* if *y* is a vertex on the path from *rt*(*T*) to *x*. If {*x*,*y*}∈*E*(*T*) and *x*≼_*T*_*y*, then *y* is the *parent* of *x* and *x* is a *child* of *y*. The *least common ancestor (LCA)* of a non-empty subset *L*⊆*V*(*T*), denoted by LCA_*T*_(*L*), is the unique smallest upper bound of *L* under ≼_*T*_.

For a rooted phylogenetic tree T=(T,φ) on *X*, let *T*_*v*_ denotes the subtree of *T* rooted at vertex *v*∈*V*(*T*). For each node $v\in I(T),C_{T}(v)$ is defined to be the set of leaf labels $\left\{\varphi (u)\in X:u\in ℒ(T_{v})\right\}$. Set $C_{T}(v)$ is called a *cluster*. Let ℋ(T) denote the set of all clusters of .

The RF distance between rooted phylogenetic trees T=(T,φ),S=(S,ϕ) on *X*, *Y*, respectively, such that *X*=*Y*, is defined as [33]

RF(T,S):=|(ℋ(T)∖ℋ(S))∪(ℋ(S)∖ℋ(T))|.

When *X*⊂*Y*, we extend the RF distance in the same way as for unrooted trees. That is, $\text{RF}(T,S):=\text{RF}(T,S_{|X})$, where $S_{|X}:=(S_{|X},\phi_{|X})$ is the rooted phylogenetic tree; here, *S*_|*X*_ is obtained from *S*[ *X*] by suppressing all non-root degree-two vertices, *ϕ*_|*X*_ is the bijective mapping $\phi_{|X}:ℒ(S_{|X})\to X$, where for each $v\in ℒ(S_{|X}),\phi_{|X}(v)=\phi (v)$.

**Figure 5:**
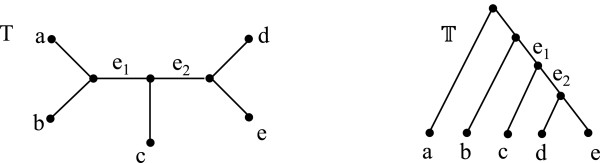
**SPR operation.** A schematic representation of the SPR operation.

Let **T** and **S** be two unrooted phylogenetic trees on *X* and *Y*, respectively, such that *X*⊆*Y*. Let  and  be the rooted phylogenetic trees that result from rooting the underlying trees of **T** and **S** at the branches incident on some arbitrarily-chosen but fixed leaf label *r*∈*X* (Figure 5). (The leaf label sets of  and  are *X* and *Y*, respectively.)

#### Lemma 1 ([30])

Let **T** and **S** be two unrooted phylogenetic trees on the same leaf label set, then RF(T,S)=RF(T,S).

We now show how to compute the RF distance between T=(T,φ) on *X* and S=(S,ϕ) on *Y*, when *X*⊆*Y*, without explicitly building $S_{|X}$. We need two concepts. Let *v*∈*I*(*S*). The *restriction* of $C_{S}(v)$ to *X* is ${Ĉ}_{T}(v):=\{w\in Y:\phi^{-1}(w)\in ℒ(S_{v})\text{and}w\in X\}.$

The *vertex function*$f_{S}$ assigns each *u*∈*I*(*T*) the value $f_{S}(u)=|U|$, where $U:=\{v\in I(S):C_{T}(u)={Ĉ}_{T}(v)\}$. Observe that if *X*=*Y*, then for all $u\in I(T),f_{S}(u)\leq 1$.

#### Lemma 2 ([30])

$\text{RF}(T,S)=|ℒ(T)|-|I(T)|+2|F_{S}|-2$, where $F_{S}:=\{u\in I(T):f_{S}(u)=0\}$.

We now describe a *O*(*n*)-time algorithm to compute the initial vertex function for  relative to , along with the RF distance between these two trees. The algorithm relies on LCAs. For  and , the *LCA mapping*${ℳ}_{S,T}:V(S)\to V(T)\cup \{\xi \}$ is defined as

$${ℳ}_{S,T}(u):=\left\{\begin{array}{ll} {\text{LCA}}_{T}(\phi^{-1}({Ĉ}_{T}(u))), & \;\text{if}\;{Ĉ}_{T}(u)\neq \phi ;\\ \xi , & \;\text{otherwise.} \end{array}\right)$$

See Figure 6.

**Figure 6:**
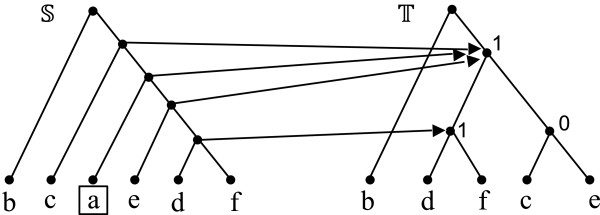
**LCA mapping.** The LCA mapping from  to . Vertex *ϕ*^-1^(*a*) in the underlying tree of  is mapped to *ξ* as *a*∉*X*. The internal vertices of the underlying tree of  are labeled with the values of the vertex function.

#### Lemma 3 ([30])

For all $u\in I(T),f_{S}(u)=|B|$, where $B:=\{v\in I(S):{ℳ}_{S,T}(v)=u$ and $\left|C_{T}(u)|=|{Ĉ}_{T}(v)|\right\}$.

The LCA computation of  is linear-time in the size of , and the LCA mapping from  to  can be done in *O*(*n*) time [37] in bottom-up manner. Further, from Lemmas 2 and 3 we can compute the RF distance between  and  in *O*(*m*) time as well.

### Solving the SPR search problem

Let T=(T,φ) be a mul-tree (on *X*) in  and S=(S,ϕ) be the input species tree (on *Y*). We now show how to compute the RF distance from  to each tree in the ${\text{SPR}}_{S}$ neighborhood in time that is linear in the size of the neighborhood. By Theorem 3, computing the RF distance between  and each $S^{\prime }\in \text{SP}R_{S}$ reduces to computing the RF distance between **T** and each **S**^′^, where **T** and **S**^′^ are the mutually consistent full differentiations of  and the extension of  relative to .

Suppose an SPR operation on  cuts the edge *e*={*x*,*y*}∈*S*, and that $\dot{x},\dot{y}$ are the subtrees of *S*-*e* containing *x*, *y*, respectively. Suppose subtree $\dot{y}$ is pruned and regrafted by the same cut edge to a new vertex obtained by subdividing an edge in $\dot{x}$. The degree-two vertex *x* is suppressed and the new vertex is denoted by *x*. Observe that there are *O*(*n*) possible edges in $\dot{x}$ to regraft $\dot{y}$. We perform regrafts in an order that leads to a constant time RF distance computation for each successive regraft.

We begin by regrafting $\dot{y}$ at an edge incident to a leaf in $\dot{x}$. Let $\bar{S}$ be the phylogenetic tree obtained from performing the prune-and-regraft. Let **T** (on *X*^′^) and $\bar{S}$ (on *Y*^′^) be the mutually consistent full differentiations of  and the extension of $\bar{S}$. We compute the RF distance between **T** and $\bar{S}$ using the algorithm described in Section Robinson-Foulds distance and least common ancestors. This method works by computing the RF distance between the rooted phylogenetic trees  and $\bar{S}$ obtained by rooting **T** and $\bar{S}$ at any leaf label in *X*^′^∩*Y*^′^. (Note that if $X\cap \phi (ℒ(\dot{x}))=\emptyset$ or $X\cap \phi (ℒ(\dot{y}))=\emptyset$, then ’s distance from $\bar{S}$ is the same as its distance from .) The algorithm also computes the LCAs for  and the LCA mapping from $\bar{S}$ to .

**Figure 7:**
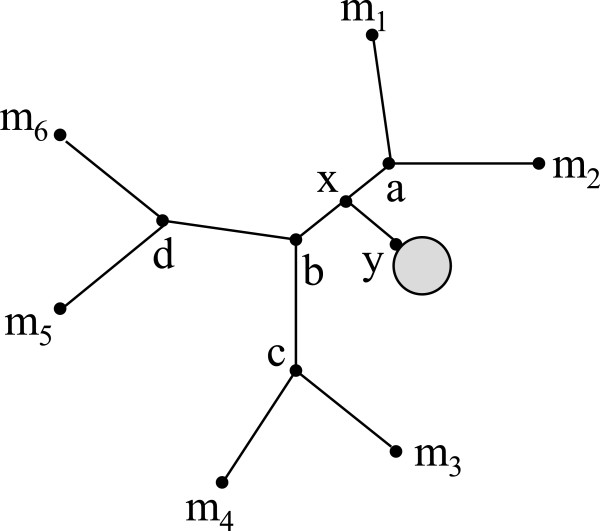
**Iterating a tree.** A phylogenetic tree with a subtree regrafted at an edge {*a*,*b*} of the underlying tree. One iteration of vertices in this tree is *m*_1_,*a*,*m*_2_,*a*,*b*,*c*,*m*_3_,*c*,*m*_4_,*c*,*b*,*d*,*m*_5_,*d*,*m*_6_,*d*,*b*,*a*,*m*_1_. The resulting ordering *ℵ* is {*m*_1_,*a*},{*a*,*m*_2_},…,{*a*,*m*_1_}.

We perform the remaining regrafts of $\dot{y}$ on edges in $\dot{x}$ by iterating through the vertices of $\dot{x}$, starting from a leaf and exploring as far as possible along each branch before backtracking. The *k*^*t* *h*^ regraft is performed on the edge between the *k*^*t* *h*^ and *k*+1^*s* *t*^ vertices in this iteration. Let us denote this ordering of edges by *ℵ*. See Figure 7. Observe that each two distinct consecutive edges in *ℵ* are adjacent. We will show that, after the initial RF distance computation for $\bar{S}$, we can compute in constant time the RF distance for the result of regrafting on each successive (adjacent) edges in *ℵ*.

Beginning with $\bar{S}$, each $S^{\prime }\in \text{SP}R_{S}$ helps in computing the RF distance of the next tree in the above regraft order. Assume that $S^{\prime }\in \text{SP}R_{S}$ results from regrafting $\dot{y}$ at edge {*a*,*b*} in $\dot{x}$, such that *x* subdivides the edge {*a*,*b*} and neighbors to vertex *y* in $\dot{y}$, as shown in Figure 7. Let the rooted phylogenetic tree obtained after extending and differentiating $S^{\prime }$ be denoted by $S^{\prime }$. The LCA mapping and RF distance have been computed for $S^{\prime }$. Let $S^{\prime \prime }\in \text{SP}R_{S}$ denote the tree obtained by regrafting $\dot{y}$ on edge {*b*,*c*} in $\dot{x}$ and the rooted counterpart of $S^{\prime \prime }$ is $S^{\prime \prime }$.

Next, we find the vertices of $S^{\prime \prime }$ whose LCA mappings have changed as a result of the SPR operation. Let *T*, *S*^′^ and *S*^′′^ be the underlying trees of $T,S^{\prime }$ and $S^{\prime \prime }$, respectively. Based on the topology of *S*^′^, there are three cases:

1. *x is parent of b and b is parent of c.* For all $t\in I(S^{\prime \prime })\setminus \{x,b\},{ℳ}_{S^{\prime \prime },T}(t)$ = ${ℳ}_{S^{\prime },T}(t)$. Further, ${ℳ}_{S^{\prime \prime },T}(b):={ℳ}_{S^{\prime },T}(x)$, and ${ℳ}_{S^{\prime \prime },T}(x):=\text{LCA}({ℳ}_{S^{\prime },T}(c),{ℳ}_{S^{\prime },T}(y))$.

2. *b is parent of c and x.* For all $t\in I(S^{\prime \prime })\setminus \{x\},{ℳ}_{S^{\prime \prime },T}(t)$ = ${ℳ}_{S^{\prime },T}(t)$. Further, ${ℳ}_{S^{\prime \prime },T}(x):=\text{LCA}({ℳ}_{S^{\prime },T}(c),{ℳ}_{S^{\prime },T}(y))$.

3. *b is parent of x and c is parent of b.* For all $t\in I(S^{\prime \prime })\setminus \{b,x\},{ℳ}_{S^{\prime \prime },T}(t)$ = ${ℳ}_{S^{\prime },T}(t)$. Moreover, ${ℳ}_{S^{\prime \prime },T}(x):={ℳ}_{S^{\prime },T}(b)$, and ${ℳ}_{S^{\prime \prime },T}(b):=\text{LCA}({ℳ}_{S^{\prime },T}(d),{ℳ}_{S^{\prime },T}(a))$.

Since we can check in constant time which one of the above three cases holds, the LCA mappings can be updated in constant time too. Let *H* be a set $\left\{u\in I(T):f_{S^{\prime \prime }}(u)\neq f_{S^{\prime }}(u)\right\}$. Observe that *H* has at most four vertices, and thus it be computed in constant time. Let *G* denote the set $\left\{w\in H:f_{S^{\prime }}(w)=0,\text{but}f_{S^{\prime \prime }}(w)\geq 1\right\}$, and *L* denote the set $\left\{w\in H:f_{S^{\prime }}(w)\geq 1,\text{but}f_{S^{\prime \prime }}(w)=0\right\}$.

#### Lemma 4

$\text{RF}(S^{\prime \prime },T)=\text{RF}(S^{\prime },T)-2|G|+2|L|$.

#### Proof

$$\begin{array}{lll} \text{RF}(S^{\prime \prime },T) & =|ℒ(T)|-|I(T)|-2+2|F_{S^{\prime \prime }}|\; & \;\\ & =|ℒ(T)|-|I(T)|-2\; & \;\\ & \;+2|\{u\in I(T):f_{S^{\prime \prime }}(u)=0\}|\; & \;\\ & =|ℒ(T)|-|I(T)|-2+2|F_{S^{\prime }}|\; & \;\\ & \;-2|\{u\in H:f_{S^{\prime }}(u)=0\&f_{S^{\prime \prime }}(u)\geq 1\}|\; & \;\\ & \;+2|\{u\in H:f_{S^{\prime \prime }}(u)=0\&f_{S^{\prime }}(u)\geq 1\}|\; & \;\\ & =\text{RF}(S^{\prime },T)-2|G|+2|L|\; & \; \end{array}$$

□

Thus, after the initial regraft of $\dot{y}$ at a leaf in $\dot{x}$, we can compute in constant time the RF-distance between  and the species tree that results from each subsequent regraft.

Next, we present the results on complexity of our algorithm. Recall that *n* is the size of the species tree and *m* is the sum of *n* and the size of an input gene tree, where all the input gene trees are considered to have approximately the same size.

#### Lemma 5

Let {*x*,*y*} be an edge of *S* and let $\dot{x}$ and $\dot{y}$ be the subtrees of *S* containing *x* and *y*, respectively, that result from deleting {*x*,*y*}. The RF distance for the set of trees obtained by regrafting $\dot{x}$ (resp. $\dot{y}$) on each edge in $\dot{y}$ (resp. $\dot{x}$) can be computed in *Θ*(*m*) time.

#### Proof

The RF distance computation for $\bar{S}$, obtained by pruning $\dot{y}$ and regrafting at a leaf in $\dot{x}$, can be done in *Θ*(*m*) time. After $\bar{S}$, the RF distance for each phylogenetic tree $S^{\prime }$ obtained by regrafting $\dot{y}$ on each edge in $\dot{x}$, can be computed in constant time by performing regrafts in the order of *ℵ*. There are *Θ*(*n*) edges in *ℵ*, thus the RF computation for all the phylogenetic trees can be done in *Θ*(*m*) time. The same argument applies for pruning $\dot{x}$ and regrafting on the edges in $\dot{y}$. □

#### Theorem 4

The SPR Search problem can be solved in *Θ*(*nmk*) time.

#### Proof

The underlying tree *S* of  has *Θ*(*n*) internal edges. For each edge {*x*,*y*} in *S*, let $\dot{x}$ and $\dot{y}$ be the subtrees of *S* defined in the statement of Lemma 5. The RF distance for all the phylogenetic trees obtained by regrafting $\dot{x}$ (or $\dot{y}$) on each edge in $\dot{y}$ (or $\dot{x}$) can be computed in *Θ*(*m*) time from Lemma 5. Thus the RF distance for *k* input mul-trees can be checked in *Θ*(*mk*) time. The total execution time for *Θ*(*n*) internal edges must be *Θ*(*nmk*). □

## Experimental evaluation

In order to evaluate the performance of the MulRF method, we implemented the heuristic algorithm of Section Solving the MulRF problem using C/C++. The MulRF software as well as simulated data sets (explained next) are freely available for download at http://genome.cs.iastate.edu/CBL/MulRF/.

### Simulated data set

#### 

##### *Methods.*

We used simulation experiments to evaluate the performance of MulRF and compare it to GTP methods. Since the MulRF method is designed for use with multi-copy gene trees, we focus on simulating genes that could have a history of duplication and/or lateral transfer. We first generated model species trees using the uniform speciation (Yule) module in the program Mesquite [38]. Two sets of model trees were generated: i) 50-taxon trees of height 220 thousand years (tyrs), ii) 100-taxon trees of height 440 tyrs. Note that the dates are relative; they do not have to represent thousands of years.

Next, we evolved 150 and 300 gene trees for each 50- and 100-taxon model species tree, respectively. For each gene tree a single gene birth node is chosen from the species tree nodes. Among all the simulated gene trees for a species tree, four gene trees have the gene birth node that is same as the root of the model tree. This represents the sampling that would result from an experiment looking at genes from a few distantly related species. The rest had a gene birth node, which is selected at random using the model species tree topology and branch lengths. Starting from the children of the root, a Poisson process is tested along the parent edge of each node. If the birth occurs, the corresponding node becomes the birth node for that gene tree. This represents the sampling that would result from a study of closely related species.

We simulated the evolution of the gene trees within the model species tree using our C++ implementation of the duplication-loss model of [39]. We applied LGT events on the evolved gene trees, using the standard subtree transfer model of LGT. One LGT event causes the subtree rooted at a vertex *c* to be pruned and regrafted at an edge (*a*,*b*), where *a* and *b* together are not in the path from the root (of the tree) to *c*. We used gene duplication and loss (D/L) rate of 0.002 events/gene per tyrs and LGT rate of 0 to 2 events per gene tree. Note that the gene tree simulations without LGT follow a molecular clock model (equal rates of molecular evolution along all branches of the gene tree), but the simulations with LGT violate the molecular clock.

We generated gene trees based on four evolutionary scenarios: i) no duplications, losses, or LGT (called *none*), ii) D/L rate 0.002 and no LGT (called *dl*), iii) no duplication or loss, and LGT rate 2 (called *lgt*), and iv) D/L rate 0.002 and LGT rate 2 (called *both*). The parameter values (evolutionary scenario and model tree size) for each simulation are called the *model condition*; 20 model species trees were generated for each model condition. We deleted 0 to 25% of leaves (selected at random) from each gene tree to represent missing data or unsampled, which is common in almost all phylogenomic studies. For each gene tree, we used Seq-Gen [40] to simulate a DNA sequence alignment of length 500 based on the GTR+Gamma+I model. The parameters of the model were chosen with equal probability from the parameter sets estimated in [41] on three biological data sets, following [42]. We estimated maximum likelihood trees from each simulated sequence alignment using RAxML [43], performing searches from 5 different starting trees and saving the best tree. Since the true root of a gene tree with possible duplication and loss often is unknown, we rooted each estimated gene tree at the midpoint of the longest leaf-to-leaf path using Retree [44] before the species tree construction.

##### *Species tree estimation.*

We estimated species trees with GTP minimizing only the number of duplications (Only-dup) [13], GTP minimizing duplications and losses (Dup-loss) [14], GTP minimizing LGT events (SPR supertree or SPRS for short) [16], and the MulRF heuristic. Both Only-dup and Dup-loss were executed with their default settings, including a fast leaf-adding heuristic for initial species tree construction. SPRS was run with 25 iterations of the global rearrangement search option. For 50-taxon data sets, it calculated the exact rSPR distance if it was 15 or less, and otherwise it estimated the rSPR distance using the 3-approximation. For the 100-taxon data sets, we used the 3-approximation of the rSPR distance. SPRS does not allow mul-trees as input. Therefore we only ran it on *none* and *lgt* data sets. Experiments were performed on the University of Florida High Performance Computing (HPC) cluster. We performed the experiments on the HPC cluster in order to simultaneously run the many simulations and phylogenetic analyses. However, all of the analyses (including SPRS, GTP, and MulRF) are sequential and easily run on a desktop machine. The running times are given in Table 1. The HPC cluster has cores of 2.3, 2.6, 2.9, or 2.66GHz on Opteron or Intel processors with 2 to 4GB RAM.

**Table 1:** Execution time

**Num. Taxa**	**Sets**	**Only-dup**	**Dup-loss**	**SPRS**	**MulRF**
50	*none*	<1s	2 s	8 h 34 m 32 s	3 s
	*lgt*	<1s	2 s	8 h 30 m 30 s	2 s
	*dl*	<1s	3 s	NA	6 s
	*both*	<1s	3 s	NA	6 s
100	*none*	9 s	37 s	21 h 34 m 25 s	58 s
	*lgt*	11 s	49 s	19 h 6 m 9 s	51s
	*dl*	9 s	30 s	NA	1 m 11 s
	*both*	11 s	37 s	NA	1 m 15 s

Running time for species tree estimations.

##### *Results.*

We report the average topological error (ATE) for each model condition. This is the average of the normalized RF distance (dividing the RF distance by number of internal edges in both trees) between each of the 20 model species trees and their estimated species trees. An ATE of 0 indicates that two trees are identical, and an ATE of 100 indicates that two trees share no common splits.

**Figure 8:**
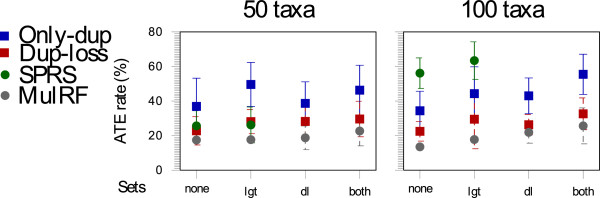
**Experimental result of simulated data.** Average topological error (means with standard error bars) for species tree constructed by Only-dup, Dup-loss, SPRS, and MulRF method, for all four model conditions.

For each set of 50- and 100-taxon model trees, the MulRF species trees are more accurate (lower ATE rate) than those produced by the other three methods. The ATE rate of MulRF is 16.75% to 39.91% lower than the method of lowest ATE rate among other three methods (Figure 8).

In order to examine how Only-dup, Dup-loss, and SPRS methods perform when gene tree simulations only include events that these methods assume to be the source of discordance, we studied the performance of Only-dup and Dup-loss on evolutionary scenario *dl* and SPRS on *lgt*. In both evolutionary scenarios we found that the ATE rate of MulRF was lowest for both 50- and 100-taxon data sets (Figure 8). Surprisingly for *lgt*, while the ATE rate of SPRS was lower than Only-dup and Dup-loss in 50-taxon, the ATE rate of the former was much higher than that of the latter two in 100-taxon data sets (Figure 8).

We also examined the accuracy of species tree estimates by Only-dup, Dup-loss, and SPRS when gene tree simulations include events that these methods do not assume to be the source of discordance (e.g., *dl* and *both* for SPRS, *lgt* and *both* for Only-dup and Dup-loss). While SPRS could not be tested on *dl* and *both* because they included mul-trees, Only-dup and Dup-loss had high ATE rate for *lgt* and *both* (Figure 8). In general, Only-dup’s estimate had much higher ATE rate compared to Dup-loss in the presence of LGT events; the ATE rate of MulRF was lowest among all the methods (Figure 8).

### Biological data set

We also tested the performance of the MulRF method on a gene tree data set from 22 plant species. These species were chosen because they are phylogenetically diverse, and they all have fully sequenced and annotated genome sequences. This makes it possible to obtain a large number of gene trees with potentially no missing sequences. Furthermore, there is much support for the relationships among most of these species (e.g., [45]), and therefore, it provides an empirical system on which we can evaluate the performance of MulRF. We obtained nucleotide alignments from gene families that had been generated from genome sequences with OrthMCL [46] and aligned with MAFFT [47]. We selected the gene family alignments that contained sequences from at least 20 of the 22 species and had a maximum of 50 gene sequences. This was a total of 1910 gene alignments. We estimated maximum likelihood trees for all of the genes using GTRCAT model in RAxML [43]. The unrooted gene trees were used as input for the MulRF heuristic. The Only-dup and Dup-loss methods require rooted input trees. Thus, we rooted all of the gene trees on the longest branch using Newick utilities [48]. This is similar to mid-point rooting, and in our experience it often provides a good starting point for input gene trees in GTP analyses. The rooted gene trees were used as input for GTP analysis using Only-dup [13] and Dup-loss [14]. Only-dup and Dup-loss were executed with the default SPR search, including a fast leaf-adding heuristic for initial species tree construction, and searching for an optimal root by re-rooting the gene trees after each SPR search (e.g., [13]; [17]). We could not run SPRS on this data set because it contains mul-trees.

**Figure 9:**
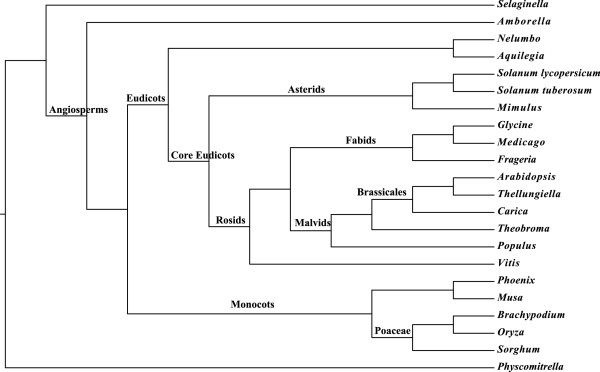
**Experimental result of biological data.** The MulRF species tree of plant gene tree data set.

The MulRF heuristic completed in 4 minutes and 4 seconds on a Mac laptop with a 2.26 GHz Intel processor and 4GB RAM. The resulting species tree is largely consistent with the most recent phylogenetic analyses (Figure 9; e.g., [45]). *Amborella* is sister to the other angiosperms and monocots and eudicots form clades. Within the eudicots there is a core-eudicot clade, and within the core-eudicots the rosid clade is sister to the asterid clade. The malvids are sister to the fabids within the rosids. Interestingly, *Populus* groups with the malvids, consistent with recent analyses of nuclear and mitochondrial, but not chloroplast, data (e.g., [49]; [17]). There are two minor differences from the generally accepted reltionships: Phoenix should be sister to Musa + Poaceae rather than sister to Musa, and Aquilegia should be sister to the other eudicots rather than Nelumbo. The ATE for the MulRF tree is 0.11. Thus, it appears that MulRF can quickly estimate a nearly accurate species trees from large-scale plant genomic data sets. The Only-dup tree heuristic completed in 7 seconds, and if we unroot the result, it is identical to the MulRF tree. The Dup-loss tree, which completed in 7 seconds, had a less accepted topology, placing *Amborella* sister to the monocots instead of sister to other angiosperms and *Vitis* sister to the asterids rather than with the rosids. The ATE for the Dup-loss tree is 0.21.

## Conclusion

We presented the new MulRF method for inferring species tree from incongruent gene trees that is based on a generalized form of the RF distance. Unlike most previous phylogenetic methods using gene trees, our approach is based on a generic tree distance measure that is not linked to any specific biological processes. As a result, it is intuitively appealing for analyses of genomic data sets, in which many processes such as deep coalescence, recombination, gene duplications and losses, and LGT, as well as phylogenetic error likely contribute to gene tree discord. In simulation experiments, the MulRF method estimated species trees more accurately than several GTP methods, and it appears to be relatively robust to the effects of phylogenetic error, gene duplication and loss, and LGT. In addition, the MulRF method is fast, estimating 100-taxon species trees from hundreds of gene trees in under two minutes and a plant data set with 22 taxa and nearly 2000 gene trees in just over 4 minutes.

Our simulation experiments greatly simplify the true processes of genomic evolution. We focused only on processes that reflect the objectives of the GTP methods, and we emphasized on duplication and loss, because that especially relevant to the evolution of multi-copy gene trees. Still, even in these conditions in which we might expect GTP to perform well, we find that MulRF obtains more accurate results than GTP in most instances. This does not mean that MulRF will always outperform GTP, but we suggest that MulRF can quickly provide an interesting alternate perspective on species tree inference. More tests are needed to characterize the performance of MulRF methods under different evolutionary scenarios.

Another future direction will be to incorporate estimates of gene tree uncertainty into the supertree analysis by weighing the splits differently when computing the RF distance. Also, the effectiveness of the MulRF method in inferring species trees from multi-copy gene trees suggests that other tree distance measures can be used in the same context. A natural candidate for study is the quartet distance. Future work should also evaluate the suitability of different distance metrics in estimating species trees under different error models and evolutionary scenarios.

## Competing interests

The authors declare that they have no competing interests.

## Authors’ contributions

RC designed and implemented the algorithm, developed the NP-completeness proof, performed simulation experiments, and wrote major parts of the paper. JGB conducted experiments on biological data set and contributed to the writing of the paper. DFB supervised the project and contributed to the writing of the manuscript. All authors read and approved the final manuscript.

## Supplementary Material

Additional file 1 **Inferring species trees from incongruent multi-copy gene trees using the Robinson-Foulds distance.** A pdf file containing the proof of Theorem 1. Click here for file
