# Supplementary material for: Inferring species trees from incongruent multi-copy gene trees using the Robinson-Foulds distance
Source: Algorithms Mol Biol. 2013 Nov 1;8:28. doi: 10.1186/1748-7188-8-28 (PMC3874668; doi:10.1186/1748-7188-8-28)
Supplement: Additional file 1 — Inferring species trees from incongruent multi-copy gene trees using the Robinson-Foulds distance. A pdf file containing the proof of Theorem 1. [file 1748-7188-8-28-S1.pdf]

Additional file 1: Inferring Species Trees from Incongruent Multi-Copy Gene Trees Using the Robinson-Foulds Distance

1 Computing the RF Distance between two mul-trees is NP-complete

The NP-completeness proof is by reduction from the following NP-complete problem [1].

Problem 1 (Exact Cover by 3-Sets (X3C))
Input: $S := \{s_1, ..., s_n\}$, where $n = 3q$, and $C := \{C_1, ..., C_m\}$ such that $C_i = \{s_{i_1}, s_{i_2}, s_{i_3}\}$.
Output: Are there exist sets $C_{i_1}, ..., C_{i_q}$ such that $\bigcup_{j=1}^{q} C_{i_j} = S$?

Note that X3C remains NP-complete even when each element of S occurs in *exactly* three subsets in C and thus $m = n = 3q$ [2]. We use this version of X3C in our reduction. For a given instance of the X3C problem, we construct two mul-trees $\mathcal{T}_1$ and $\mathcal{T}_2$ on the same set of labels and with matching label multiplicities, such that transforming $\mathcal{T}_1$ into $\mathcal{T}_2$ (or vice versa) requires κ (to be specified later) contractions and refinements if and only if an exact cover of S exists.

Mul-trees $\mathcal{T}_1$ and $\mathcal{T}_2$ are constructed in the following way. For each $s_i \in S$, we construct two rooted, binary singly-labeled trees $\mathbb{T}$ and $\mathbb{T}'$ on the same set of labels that take a "large" number of contractions and refinements to transform into each other (see Fig. 1). Let k and t be two positive integers such that $k + 2 \geq n^2$ and $k + 2 = 2^t$; $\mathbb{T}$ and $\mathbb{T}'$ are on the same $(k + 2)$-element leaf label set. $\mathbb{T}'$ has the same underlying tree as $\mathbb{T}$ but different labeling map. In particular, for each cherry[1] (x, y) in $\mathbb{T}$, x and y are in different clusters $C_{\mathbb{T}'}(u)$ and $C_{\mathbb{T}'}(v)$ in $\mathbb{T}'$, where u and v are two children of the root in the underlying tree of $\mathbb{T}'$. Both $\mathbb{T}$ and $\mathbb{T}'$ have unique leaf labels for each $s_i \in S$.

Lemma 1. $RF(\mathbb{T}, \mathbb{T}') = 2k$.

Proof. $RF(\mathbb{T}, \mathbb{T}') = 2|\mathcal{H}(\mathbb{T}) \backslash \mathcal{H}(\mathbb{T}')|$, since $\mathbb{T}$ and $\mathbb{T}'$ are binary singly-labeled trees. Further, since $\mathbb{T}$ and $\mathbb{T}'$ are on the same set of $k + 2$ labels, $\mathcal{H}(\mathbb{T}) = \mathcal{H}(\mathbb{T}') = k$. Thus, it suffices to show that no cluster in $\mathbb{T}$ matches any cluster in $\mathbb{T}'$, but this follows directly from the construction. □

[1] In a phylogenetic tree $\mathcal{T} = (T, \phi)$ on X, a pair of leaf labels (a, b) forms a cherry if $\phi^{-1}(a)$ and $\phi^{-1}(b)$ are adjacent to the same internal vertex in T.

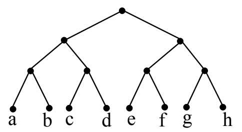

Figure 1: Two possible singly-labeled trees $\mathbb{T}$ and $\mathbb{T}'$ on an 8-element label set. The RF distance between $\mathbb{T}$ and $\mathbb{T}'$ is 12.

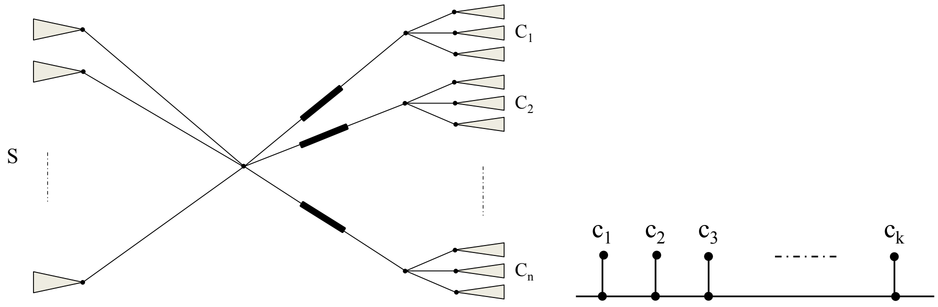

(a) (b)

Figure 2: (a) Structure of mul-tree $\mathcal{T}_1$ and (b) a toll sequence of k leaves.

We now describe the construction of $\mathcal{T}_1$ and $\mathcal{T}_2$. Let $n = 3q$. Figure 2(a) outlines the structure of $\mathcal{T}_1$. The solid rectangles represent *toll* sequences of k uniquely labeled leaves. The toll sequence is a caterpillar tree, where leaf vertices of both ends are in fact the two internal vertices (the center vertex of degree $2n$ and degree-four vertex corresponding to a C_i ($1 \leq i \leq n$)) in the underlying tree of $\mathcal{T}_1$ (Fig. 2(b)). The left side of $\mathcal{T}_1$ has n triangles, one for each of the n elements in S. Each triangle represents a phylogenetic tree $\mathbb{T}$ corresponding to each $s_i \in S$, connecting through its root. The right side of $\mathcal{T}_1$ has n sets of three triangles corresponding to the subsets in C; for each subset $C_i = \{s_{i_1}, s_{i_2}, s_{i_3}\}$, the triangles represent three phylogenetic trees $\mathbb{T}'$s, corresponding to each s_{i_j} ($1 \leq j \leq 3$), connected through their roots.

The structure of mul-tree $\mathcal{T}_2$ is similar to that of $\mathcal{T}_1$, except that $\mathcal{T}_2$ has $\mathbb{T}'$ for each $s_i \in S$ and $\mathbb{T}$ for each element of $C_i \in C$ ($1 \leq i \leq n$). Thus, $\mathcal{T}_2$ has $\mathbb{T}'$s on the left side and $\mathbb{T}$s on the right side, the opposite of $\mathcal{T}_1$.

Lemma 2. *$\mathcal{T}_1$ and $\mathcal{T}_2$ can be constructed in polynomial time.*

Proof. $\mathbb{T}$ and $\mathbb{T}'$ have the leaf label set of $k + 2$ elements. Both $\mathbb{T}$ and $\mathbb{T}'$ can be constructed in polynomial time, and so can their $8n$ copies ($4n$ for $\mathcal{T}_1$ and $4n$ for $\mathcal{T}_2$). Further, the $2n$ toll sequences (n for $\mathcal{T}_1$ and n for $\mathcal{T}_2$) can be constructed in polynomial time. The number of remaining vertices in $\mathcal{T}_1$ and $\mathcal{T}_2$ is constant. □

The connection between exactly covering S and transforming $\mathcal{T}_1$ into $\mathcal{T}_2$ by contractions and refinements is as follows: To transform $\mathcal{T}_1$ into $\mathcal{T}_2$, we need to convert each $\mathbb{T}$ on the left into $\mathbb{T}'$ and each $\mathbb{T}'$ on the right into $\mathbb{T}$. From Lemma 1, this costs $24qk$ contractions and refinements. A rather clever technique is to swap $3q$ $\mathbb{T}$s on the left with their counterparts on the right and to transform the remaining $6q$ $\mathbb{T}'$s on the right into $\mathbb{T}$s. If an exact cover $C_{i_1}, ..., C_{i_q}$ of S exists, we can partition

the $3q$ $\mathbb{T}$s into q groups according to the cover. For each C_j ($j = i_1, ..., i_q$) in the cover, we swap the corresponding group of phylogenetic trees for sequences $s_{j_1}, s_{j_2}, s_{j_3}$ with their counterparts.

Lemma 3. *All $\mathbb{T}'$s for each C_j ($j = i_1, ..., i_q$) can be swapped with corresponding $\mathbb{T}$s by $2(k+1)$ contractions and refinements.*

Proof. Take the toll sequence corresponding to C_j and contract its $k+1$ edges; i.e., $(k-1)$ internal edges and 2 edges at both the sides of the toll sequence. Now refine it so that the corresponding $\mathbb{T}$s move in C_j and the $\mathbb{T}'$s stay on the left. This takes $2(k+1)$ contractions and refinements. □

From Lemma 3, if an exact cover of S exists, then $6q$ phylogenetic trees can be transformed by $2q(k+1)$ contractions and refinements. The remaining $6q$ $\mathbb{T}'$s can be transformed into $\mathbb{T}$s by $12qk$ contractions and refinements. Let $\kappa = 2q(k+1) + 12kq$. We have the next lemma.

Lemma 4. *If set S has an exact cover, then RF$(\mathcal{T}_1, \mathcal{T}_2) = \kappa$.*

Lemma 5. *If set S has no exact cover, then RF$(\mathcal{T}_1, \mathcal{T}_2) > \kappa$.*

Proof. Observe that to transform the $6q$ $\mathbb{T}'$s, in the right side of $\mathcal{T}_1$ into their respective $\mathbb{T}$s, we need at least $\kappa_2 = 12kq$ contraction and refinements, whether or not there is an exact cover. We claim that if S has no exact cover, then the number of additional contractions and refinements required, which we denote by κ_1, is greater than $2q(k+1)$.

If set S has an exact cover, then the $3q$ $\mathbb{T}$s on the left and the $3q$ $\mathbb{T}'$s on the right side of $\mathcal{T}_1$ can be converted into their counterparts by one of the two ways:

- **Swapping more than q triplets.** Let $q + \sigma$ triplets cover all elements in S (with some repeated elements). Now swapping $3q$ $\mathbb{T}$ with corresponding $\mathbb{T}'$ in $q + \sigma$ triplets will require $2(q+\sigma)(k+1)$ contractions and refinements. Thus, $\kappa_1 = 2(q+\sigma)(k+1)$.

- **Swapping q triplets.** Let $C_{i_1}, ..., C_{i_q}$ be the best q triplets that cover all but σ elements in S. Swapping these q triplets only converts $n - \sigma$ $\mathbb{T}$s and $\mathbb{T}'$s into their counterparts. Rest 2σ $\mathbb{T}$s and $\mathbb{T}'$s need to be converted manually. Thus, $\kappa_1 = 2q(k+1)$ + <2σ manual conversions>.

Both the above ways yield a $\kappa_1 > q(k+1)$. The total number of contractions and refinements is $\kappa_1 + \kappa_2$, which is greater than κ. □

We have the next theorem.

Theorem 1. *Set S has an exact cover if and only if RF$(\mathcal{T}_1, \mathcal{T}_2) = \kappa$.*

References

[1] M. R. Garey and D. S. Johnson. *Computers and Intractability: A guide to the theory of NP-completeness*. W. H. Freeman, New York, 1979.

[2] G. Hickey, F. Dehne, A. Rau-Chaplin, and C. Blouin. SPR distance computation for unrooted trees. *Evolutionary Bioinformatics*, 4:17–27, 2008.
